# Psychosocial Interventions for the Caregivers of patients with Epilepsy

**DOI:** 10.1192/j.eurpsy.2025.1491

**Published:** 2025-08-26

**Authors:** V. Patil

**Affiliations:** 1 Neuropsychiatry, AIIMS New Delhi, New Delhi, India

## Abstract

**Introduction:**

Epilepsy, one of the most common neurological disorders, affects around 50 million people worldwide. It is unpredictable, intrusive illness that impacts not only the patients but also those who care for them. Caregivers are vulnerable to great burden; depressive and psychosomatic symptoms, as well as physical, emotional, and economic pressures.

**Objectives:**

To explore the psychiatric comorbidities, attributes related to caregiver burden and psychosocial intervetions available to allivate the burden in **Caregivers of patients with Epilepsy**.

**Methods:**

A narrative review of the relevant studies focusing on psychiatric comorbidities and psychosocial interventions for reducing the caregiver burden in caregivers of patient with epilepsy was comducted.

**Results:**

Caregivers of patient with epilpsy have poor quality of life and are at risk of developing psychiatric illnesses. Caregiving was reported to negatively impact one’s physical and mental health, overall family functioning, and financial status. Psychological interventions such as **psychoeducation, individual, group or family counselling, Interpersonal and social support networks, relaxation therapy and cognitive behaviour therapy** have been used to treat caregiver burden associated with epilepsy caregiving.

**Conclusions:**

Caring for patients with epilepsy is challenging and it is associated with enormous burden. It can lead to mental health problems which ultimately affects the compliance to treatment and overall prognosis. Psychosocial interventions can prepare caregivers for a better role of caregiver and better management of the care process. There is increased need to focus on this unexplored area through research and to provide effective interventions as a part of clinical services.

**Disclosure of Interest:**

None Declared

